# The Effects of the COVID-19 Environments on Changes in Body Composition in Japanese Elite Fencing Athlete

**DOI:** 10.3390/sports9070095

**Published:** 2021-06-25

**Authors:** Jun Yasuda, Emi Kondo, Eri Takai, Nobuhiko Eda, Yasuyuki Azuma, Keiko Motonaga, Michiko Dohi, Akiko Kamei

**Affiliations:** 1 Japan Institute of Sports Sciences, Tokyo 115-0056, Japan; emik38113@gmail.com (E.K.); eri.takai@jpnsport.go.jp (E.T.); n-eda@dokkyomed.ac.jp (N.E.); yasuyuki.azuma@jpnsport.go.jp (Y.A.); keiko.motonaga@jpnsport.go.jp (K.M.); michiko.dohi@jpnsport.go.jp (M.D.); akiko.kamei@jpnsport.go.jp (A.K.); 2 Japan Society for the Promotion of Science, Tokyo 102-0083, Japan; 3 Faculty of Health and Sport Sciences, University of Tsukuba, Ibaraki 305-8574, Japan; 4 Department of Fundamental Education, Dokkyo Medical University, Tochigi 321-0293, Japan; 5 Medical Committee, Federation Japonaise d’Escrime, Tokyo 160-0013, Japan

**Keywords:** Olympics, fat-free mass, fat mass, coronavirus, lockdown, fencers, Japan

## Abstract

The Japanese government declared a state of emergency from 7 April to 25 May to limit people’s movement due to the coronavirus disease (COVID-19) pandemic. This pandemic negatively affects athletes’ body composition due to inactivity. Therefore, we compared the body composition data (i.e., fat-free mass (FFM) and fat mass (FM)), of 43 Japanese elite fencers (22 men, 21 women), in September 2019 for baseline, and of 21 (12 men, 9 women) who completed the following measurements in June 2020 (POST; immediately after rescinding the emergency state) and September 2020 (POST-4M; 4-months after rescinding the emergency state). Results at baseline indicate no significant differences in body compositions among fencing disciplines. We also confirmed no significant changes in body mass during the 1-year investigation period in either sex. There were no time-course changes in men’s FFM and FM; however, time-course changes in women’s FM were observed. Compared to the baseline, FM values were significantly higher at POST and then returned to baseline levels at POST-4M in women. In conclusion, the 2-month stay-at-home period due to COVID-19 negatively affected women’s FM changes, but not their FFM or men’s FM.

## 1. Introduction

The Tokyo 2020 Olympics has been postponed and re-scheduled to the summer of 2021 due to the coronavirus disease-19 (COVID-19). First reported in China, the severe acute respiratory syndrome, coronavirus-2 (SARS-CoV-2), has been spreading worldwide since December 2019. In Japan, patients with COVID-19 were first diagnosed on 16 January 2020. Consequently, the Japanese government declared a state of emergency from 7 April to 25 May to limit people’s movement. This restricted Japanese elite athletes’ training and practice, as they could not use specific practicing areas, such as gymnasiums, training gyms, or inside track and fields during these two months. Therefore, this may have caused inactivity and negatively affected athletes’ body composition.

In terms of the positive association between muscle mass and muscle strength [1,2], higher fat-free mass (FFM) can be considered one of the factors that determines athletes’ performance. For example, a cross-sectional study reported that muscle mass was positively associated with athletic performance (e.g., vertical jump or 30-m sprint time) in collegiate soccer athletes [1]. In addition, another cross-sectional study confirmed that fat mass (FM)% was negatively correlated with fencers’ performance (fencing lunge time and shuttle test), meaning that FFM% was a positive factor for better performance in Greek elite fencers [3]. Additionally, an intervention study in an untrained population demonstrated that changes in muscle volume were positively correlated with muscle strength [4]. Those studies suggested that monitoring body composition data is essential for maintaining and improving athletes’ performance.

The COVID-19 quarantine has been reported to increase FM and body mass (BM), and decrease sprint and countermovement jump abilities in Brazilian professional soccer athletes compared to traditional off-season values [5]. Owing to there being less training and practice than usual, there may be detrimental effects on body composition (e.g., FFM and FM). This problem can also occur in athletes of other sports. However, information on the effects of COVID-19 on body composition is lacking.

Therefore, the purpose of this study was to confirm the FFM and FM data on Japanese elite fencers, whom we followed in three situations: non-emergency state (baseline; September 2019), after rescission of the emergency state (POST; June 2020), and the 4-month recovery from POST (POST-4M; September 2020). We hypothesized that the 2-month emergency state negatively affected the body composition of Japanese elite fencers.

## 2. Materials and Methods

### 2.1. Subjects

Baseline data were collected from 43 fencing athletes who were candidates for the Tokyo 2020 Olympics. Of them, 22 athletes were male (mean age, 26.4 years; height, 178.4 cm; BM, 73.0 kg) and 21 were female (mean age, 24.7 years; height, 164.1 cm; BM, 57.8 kg), from three fencing disciplines: epee (male = 8; female = 6), saber (male = 7; female = 7), and foil (male = 7; female = 8). The data of 21 fencing athletes (12 men and 9 women) who underwent all the measurements at baseline, POST, and POST-4M: epee (male = 3; female = 2), saber (male = 6; female = 4), and foil (male = 3; female = 3) were analyzed as main results in this study. Table 1 shows the schedule of the measurements, including the main competitive events in Japanese fencing athletes.

The Institutional Ethics Committee of Japan Institute of Sports Sciences (No. 2021-010) approved this study and use of an opt-out consent method to obtain informed consent for data use and publication, based on the low risk (no intervention or invasiveness) to the subjects and the data already collected before conducting the study. The information explaining this study to the athletes was presented using a website (https://www.jpnsport.go.jp/hpsc/business/ourwork/tabid/1322/Default.aspx; accessed on 24 May 2021).

### 2.2. Anthropometric Measurements

Athletes’ heights were measured barefoot while wearing a swimsuit or t-shirts and shorts, using an automatic stadiometer (A & D Co. Ltd., Tokyo, Japan) at baseline. At POST and POST-4M, we did not measure height and limited measurements as much as possible to avoid COVID-19 infection. BM and body composition (FM and FFM) were assessed using bioimpedance methods (InBody 730, InBody Japan, Tokyo, Japan), and each subject washed their hands and the soles of their feet using the InBody sanitizing tissue. During the body composition assessment, the subjects were asked to ensure their arms were not touching their bodies and remain still until the assessment was finished. After one subject finished, InBody 730 was cleaned using >70% ethanol. In addition, intra-class correlation coefficients (ICC) and CV% in the measurements were confirmed as follows: Height, ICC: 0.999 (95% CI: 0.997–0.999) and CV%: 5.0%; BM, ICC: 0.975 (95% CI: 0.950–0.989) and CV%: 13.6%; BMI, ICC: 0.893 (95% CI: 0.797–0.951) and CV%: 6.6%; FFM, ICC: 0.986 (95% CI: 0.972–0.994) and CV%: 18.5%; FM, ICC: 0.792 (95% CI: 0.627–0.901) and CV%: 26.8%; and FM%, ICC: 0.899 (95% CI: 0.807–0.954) and CV%: 34.9%.

### 2.3. Statistics

Data are expressed as the mean ± SD. For baseline data comparisons, we compared the body compositions among fencing disciplines (epee, saber, and foil) using one-way analysis of variance (ANOVA) with post hoc independent *t*-tests (Bonferroni corrected). A repeated one-way ANOVA was used for time differences of each sex. Significant time effects were followed using Bonferroni corrected paired *t*-tests, and the statistical significance was set at *p* < 0.05. All statistical analyses were performed using SPSS version 24 (IBM Corp., Armonk, NY, USA). Gender comparisons were analyzed using independent *t*-tests.

## 3. Results

### 3.1. Body Composition of Athletes among Three Fencing Disciplines at Baseline

The body composition data of the 43 fencing athletes at baseline are shown in Table 2 (male) and Table 3 (female). There were no significant differences in body composition variables among the fencing disciplines (epee, saber, and foil) in men. In women, a significant difference was observed only in FFM, while there were no significant differences in the other variables. After a post hoc test for FFM in women, no significant differences were confirmed among the fencing disciplines.

### 3.2. Time-Course Changes in Body Compositions within Gender

In male athletes, there were no significant time differences in any of the body composition variables (Table 4). In contrast, there were significant time differences in FM and FM% in female athletes (Table 5). After post hoc tests, FM and FM% were significantly increased at POST compared to baseline, and decreased at POST-4M compared to POST in female athletes.

## 4. Discussion

Using the baseline data of 43 Japanese elite fencers, we examined body composition data regarding fencing disciplines. Thereafter, we examined the effect of the COVID-19 pandemic on the changes in body composition in 21 Japanese elite fencers. There were no significant differences in body composition regarding fencing disciplines in Japanese elite fencers at baseline. However, the pandemic environment in Japan increased FM only among female athletes. After rescinding the state of emergency, a 4-month recovery period reversed the negative effects on body composition; FM recovered to the baseline level in female athletes.

To the best of our knowledge, this is the first study to confirm the effects of the COVID-19 environment on body composition in elite fencing athletes. A previous study reported that changes in the elbow flexor muscle volume were significantly correlated with changes in elbow flexor muscle strength (isometric maximum voluntary force and 1-repetition maximum) in healthy young men, but not in athletes [4]. In addition, a previous study confirmed that increase in sport-specific performance was accompanied by lean body mass gains in Olympic weightlifters [6]. These indicate that the detrimental effects on body composition could be connected to a reduction in the fencer’s performance in the present study. One case report confirmed that a 2-month lockdown due to COVID-19 significantly reduced countermovement jump and squat 1-repetition maximum in elite badminton athletes in Spain [7]. In addition, 6 to 8 weeks of retraining was reported to return the two performances to similar values at baseline in this case report [7]. Considering the 2-month state of emergency period in the present study, the changes in fencer’s performance in our athletes could be similar to the results in that case report [7]. Unfortunately, to avoid COVID-19 infection, we could not assess the fencer’s performance in our population. Therefore, further studies are still needed to confirm the effect of COVID-19 environments on both fencer’s performance and body composition.

No changes in BM and FM% between the in-season and off-season periods were reported in British elite fencers [8]. However, the 2-month stay-at-home period negatively impacted body composition in this present study. Our findings support a previous study demonstrating greater detrimental effects on body composition during the COVID-19 quarantine period than the off-season period [5]. The emergency state in Japan required the whole nation to stay in their houses as much as possible, resulting in moderate to high inactivity. Although athletes’ inside activity areas were limited during the emergency state period in Japan, they could exercise outside areas, such as parks, outdoor tracks, and fields. In Spain (a 100-day lockdown), only about 5% of 544 athletes could train as usual during the lockdown [9]. In South Africa (a 35-day lockdown), 75% of the 692 athletes reported that their training had reduced during the lockdown [10]. These reports suggest that in countries where the government declared total lockdown, the detrimental effects on body composition could be equal to or more serious than those in Japan. Future studies are required to examine the relationship between the COVID-19 environment and body composition data.

Detraining studies can be one of the options for effective recovery from detrimental effects such as the state of emergency or lockdowns. In healthy young adults, a 4-month resistance training intervention has been reported to increase lean mass, and a 4-month detraining significantly reduces training-induced gains [11]. This could explain the negative changes in body composition during the 2-month stay-at-home period and the 4-month recovery in our study. In addition, in the aforementioned study [11], 1/9 volume dose training robustly retained the lean mass gains induced by the 16-week resistance training during the entire 32-week training period. Moreover, a randomized clinical trial reported that a 6-week training/3-week detraining cycle for 24 weeks resulted in similar muscle fiber gains as the continuous training program (3 days/week for 24 weeks) in healthy young men [12]. Thus, these strategies can be useful for athletes during pandemic environments, such as COVID-19, to retain or even improve their body composition.

This study has some limitations. First, we did not assess dietary intake or physical activity status during the observational period. Dietary intake, especially energy [13] and protein intake [14], have been reported to affect body composition changes with training interventions. Thus, future investigations should include information on dietary intake and physical activity states to examine the effects of pandemic environments on body composition changes. Next, the fencers in this study were not asked to standardize their meal conditions before the measurements. Although national-level athletes in Japan are well informed not to change their usual diets before body composition measurements, the variability of body contents (especially body water) could still affect the data in this study. Finally, the data of height at POST and POST-4M were self-reported values assessed at the latest measurements before POST and POST-4M as we limited the measurements to avoid COVID-19 infection. Although the fencers in this study had been speculated to have remained unchanged during the 1-year investigation period, considering their age (≥18 years), we could not completely deny the changes in height. We confirmed that ICC and CV% of height were 0.999 and 5.0%, respectively, indicating the height during the investigation period unchanged. 

## 5. Conclusions

In Japanese elite fencers, the 2-month stay-at-home period due to COVID-19 negatively affected female fencers’ body composition. Moreover, fewer detrimental effects were observed on the changes in body composition of male athletes compared to female athletes. Alternatively, the body compositions recovered 4 months after the end of the state of emergency. This may be as the athletes could resume training with coaches, trainers, and dietitians immediately after the state of emergency ended. For practical implication, constant monitoring of body composition data is essential for maintaining or improving athletes’ performances, especially under challenging situations that force them to be inactive. Additionally, the strategy of retraining or home-based training should be well-planned to countervail against the inactiveness, such as pandemic environments.

## Figures and Tables

**Table 1:** Time schedule of the measurements including main competitive events.

		2019			2020								
		Oct	Nov	Dec	Jan	Feb	Mar	Apr	May	June	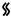	Sep	Oct
Men	Epee	M (baseline)	WC		WC GP	WC	GP	Emergency period		M (POST)		AJC	M (POST-4M)
	Saber		WC		GP	WC	WC					AJC	
	Foil		WC	WC	WC	WC GP						AJC	
Women	Epee		WC		WC GP	WC	GP					AJC	
	Saber		WC	WC	GP		WC					AJC	
	Foil		WC	WC	WC	WC GP						AJC	

Abbreviations: AJC, all Japanese cup; GP, grand prix; M, measurements; and WC, world cup.

**Table 2:** Differences in body composition of male athletes among fencing disciplines at baseline.

	Epee (*n* = 8)			Saber (*n* = 7)			Foil (*n* = 7)			*p* Values
Age (year)	25.8	±	4.5	28.1	±	5.9	25.3	±	4.4	0.517
Height (cm)	178.6	±	5.9	176.8	±	4.3	179.8	±	1.9	0.457
BM (kg)	69.9	±	5.2	74.3	±	6.1	75.3	±	4.5	0.130
BMI (kg/m^2^)	21.9	±	1.7	23.7	±	1.3	23.3	±	1.4	0.066
FFM (kg)	61.7	±	3.0	65.6	±	5.8	64.3	±	2.6	0.182
FM (kg)	8.1	±	2.5	8.7	±	0.9	11.0	±	3.0	0.076
FM%	11.5	±	2.9	11.8	±	1.2	14.5	±	3.2	0.077

Values are expressed as mean ± SD. Abbreviations: BM, body mass; BMI, body mass index; FFM, fat-free mass; and FM, fat mass.

**Table 3:** Differences in body compositions of female athletes among fencing disciplines at baseline.

	Epee (*n* = 6)			Saber (*n* = 7)			Foil (*n* = 8)			*p* Values
Age (year)	26.5	±	5.2	25.0	±	3.8	23.1	±	3.6	0.341
Height (cm)	167.1	±	4.7	163.8	±	3.8	162.1	±	5.2	0.161
BM (kg)	60.2	±	3.6	59.9	±	5.3	54.3	±	5.7	0.066
BMI (kg/m^2^)	21.6	±	1.1	22.3	±	1.6	20.6	±	1.2	0.077
FFM (kg)	47.5	±	3.0	47.6	±	2.8	42.9	±	4.4	0.031
FM (kg)	12.7	±	2.4	12.3	±	3.4	11.4	±	2.4	0.917
FM%	21.1	±	3.6	20.3	±	4.1	20.9	±	3.3	0.919

Values are expressed as mean ± SD. Abbreviations: BM, body mass; BMI, body mass index; FFM, fat-free mass; and FM, fat mass.

**Table 4:** Time-course changes in body composition of male athletes.

	Baseline			POST			POST-4M			*p* Values
Height	178.9	±	5.2	178.7	±	4.9	178.9	±	5.1	-
BM	72.9	±	5.7	73.1	±	7.3	73.8	±	7.3	0.343
BMI	22.8	±	1.4	22.9	±	1.9	23.0	±	1.7	0.480
FFM	64.2	±	4.9	63.8	±	5.6	64.9	±	6.3	0.134
FM	8.7	±	1.3	9.4	±	2.7	9.0	±	2.3	0.461
FM%	11.9	±	1.4	12.7	±	2.9	12.1	±	2.7	0.521

Values are expressed as mean ± SD. *n* = 12. Abbreviations: BM, body mass; BMI, body mass index; FFM, fat-free mass; and FM, fat mass.

**Table 5:** Time-course changes in body composition of female athletes.

	Baseline			POST			POST-4M			*p* Values
Height	164.3	±	4.0	164.2	±	4.0	164.3	±	3.9	-
BM	59.1	±	3.9	59.6	±	4.2	58.7	±	4.2	0.280
BMI	21.9	±	0.9	22.1	±	1.0	21.7	±	1.1	0.216
FFM	46.6	±	4.1	45.5	±	4.0	46.3	±	4.1	0.039
FM	12.5	±	2.1 ^a^	14.2	±	1.6 ^b^	12.4	±	2.1 ^a^	0.002
FM%	21.2	±	3.6 ^a^	23.8	±	2.8 ^b^	21.2	±	3.5 ^a^	0.001

Values are expressed as mean ± SD. *n* = 9. ab: different letters indicate significant differences (Bonferroni corrected). Abbreviations: BM, body mass; BMI, body mass index; FFM, fat-free mass; and FM, fat mass.

## Data Availability

The data are not publicly available due to privacy restrictions.
